# Acantholytic plaque on the right breast

**DOI:** 10.1016/j.jdcr.2026.04.014

**Published:** 2026-04-16

**Authors:** Trimane T. McKenzie, Ricardo White, Alicia McNish, Stephanie Smith-Matthews, Jonathan D. Ho

**Affiliations:** a Division of Dermatology, Department of Medicine, The University of the West Indies, Jamaica, West Indies; b Department of Pathology, The University of the West Indies, Jamaica, West Indies

**Keywords:** acantholytic, acantholysis, breast, Paget disease, skin of color

## Case presentation

**Fig 1:**
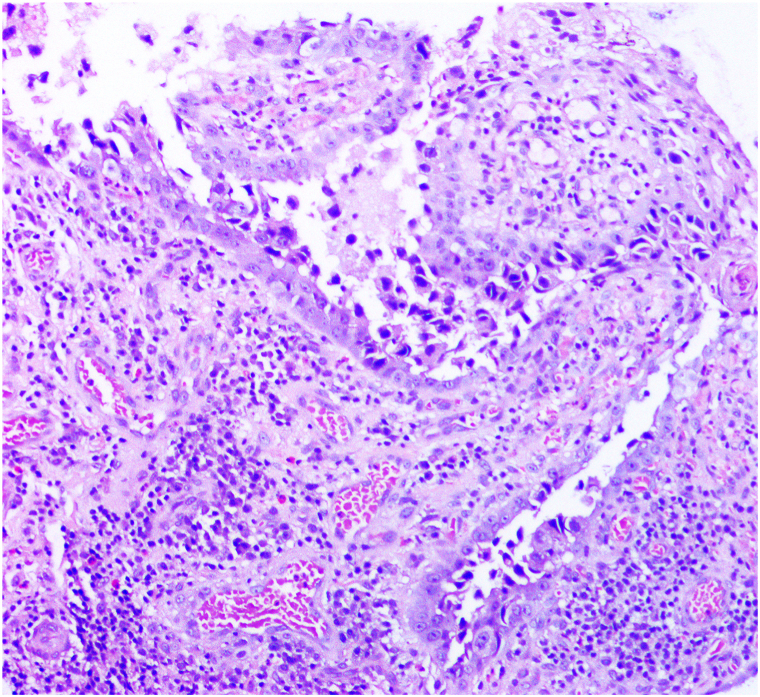
Biopsy from right areola demonstrating suprabasal acantholysis and a superficial perivascular lymphoplasmacytic infiltrate with occasional eosinophils.

A 57-year-old Afro-Caribbean female presented to dermatology for evaluation of an erosive plaque to the right breast, present for 9 months She reported a history of “stripping” to the right nipple with itching and redness. There was no history of trauma and no similar lesions in family members. The patient had a history of hypertension and an ischemic stroke 2 years prior to presentation, managed with clopidogrel, atorvastatin, amlodipine and aspirin. She reported no changes to her medications over the last 6 months. A biopsy performed at another institution prior to presentation reported erosion, suprabasal acantholysis and a superficial perivascular infiltrate of lymphocytes, eosinophils and neutrophils (Fig 1). Examination revealed an erosive plaque involving the right nipple-areolar complex with surrounding hyperpigmentation. No discernible mass was identified.

### Question 1: Which of the following is responsible for the microscopic findings seen?

**A.** Localized autoantibodies against desmoglein 3
**B.** Epidermotropic malignant cells
**C.** Mutations in *ATP2C1* gene
**D.** Mutations in *ATP2A2* gene
**E.** Chronic infection with a double-stranded DNA virus

### Answer discussion

The correct answer is B.

**Fig 2:**
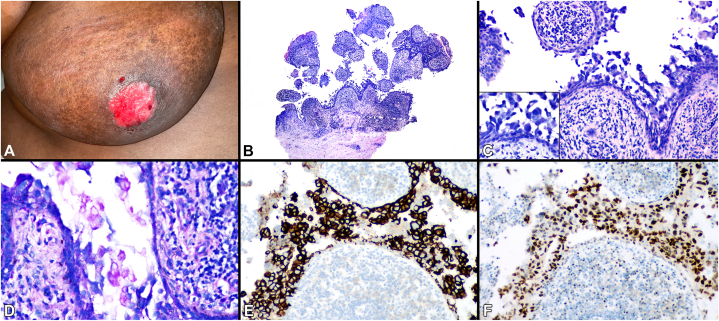
Acantholytic mammary Paget disease: Note a large ulcerated plaque replacing the nipple-areola complex **(A)**. At low power, a repeat biopsy demonstrates striking dyscohesion and suprabasal acantholysis **(B, C)**. In addition to acantholysis, there is obvious anaplasia and marked cytologic atypia compared with that seen in the previous biopsy **(C, inset)**. The acantholytic cells are positive with PAS **(D)**, CK7 **(E),** and GATA3 **(F)**, consistent with acantholytic mammary Paget disease.

This patient has the rarely described acantholytic mammary Paget disease (AMPD). Re-biopsy of the lesion after clinical examination (Fig 2) revealed similar acantholysis with more marked cytologic atypia (acantholytic anaplastic Paget disease). The cells were PAS+, CK7+ and GATA3+, consistent with AMPD (Fig 2D-F). Subsequent mammogram revealed a Breast Imaging-Reporting and Data System (BI-RADS) 3 lesion (“probably benign”), but, given the findings in the skin, image-guided breast biopsy revealed invasive ductal carcinoma.

Paget disease of the breast is a rare manifestation of breast carcinoma characterized by epidermotropic malignant cells of the nipple–areolar complex. Clinically, it typically presents as a chronic eczematous-like dermatitis, ulcerated plaques or abnormal nipple discharge.^1^

Several histological patterns of Paget disease have been described, including adenocarcinoma-like (classic), anaplastic, spindle cell, pigmented, and, rarely, acantholytic variants.^2^ The histopathologic appearance of AMPD may lead to diagnostic confusion with true acantholytic dermatoses particularly localized pemphigus vulgaris and heritable causes. Clinical context, lack of direct immunofluorescence positivity and atypical cytology help distinguish benign entities. Importantly, although most reported AMPD cases describe severe anaplasia, our case highlights that, focally, atypia may be subtle. When anaplasia is readily apparent, the differential diagnosis includes acantholytic squamous cell carcinoma *in situ* (SCCIS) and melanoma. Paget cells are typically PAS/CK7/GATA3 positive, distinguishing it from both SCCIS and melanoma. While most frequently described in mammary cases, extramammary acantholytic Paget disease has also been reported.^3^ Acantholysis in tumors such as squamous cell carcinoma is thought to occur due to the loss or reduced function of intercellular adhesion proteins, including desmosomes.^4^ Currently, no definitive pathomechanism has been described for AMPD.

The majority of patients diagnosed with mammary Paget disease have underlying invasive ductal breast cancer (∼95%).^5^ Our patient was ultimately diagnosed with invasive carcinoma of the breast with right axillary lymph node metastasis. She is currently undergoing oncologic management with systemic chemotherapy and adjuvant radiation therapy. Familiarity with unusual histopathologic subtypes prevent delay in diagnosis (Fig 2).

## Conflicts of interest

None disclosed.
